# Tetramethyl Cucurbit[6]uril–Porphyrin Supramolecular Polymer Enhances Photosensitization

**DOI:** 10.3390/ijms252313037

**Published:** 2024-12-04

**Authors:** Bo Xiao, Yueyue Liao, Jinyu Zhang, Ke Chen, Guangwei Feng, Jian Feng, Chunlin Zhang

**Affiliations:** School of Basic Medical Sciences/School of Medical Humanities, Guizhou Medical University, Guiyang 550025, China; xiaobogzmu@163.com (B.X.); huchuanlyy@163.com (Y.L.); zjy08022024@163.com (J.Z.); clinmed_kechen@163.com (K.C.); fengguangwei@gmc.edu.cn (G.F.)

**Keywords:** tetramethyl cucurbit[6]uril, porphyrin, supramolecular polymer, photosensitizer, photodynamic therapy

## Abstract

Porphyrins serve as photosensitizers (PS) in the realm of cancer photodynamic therapy (PDT). Upon excitation by laser light, porphyrins are capable of converting molecular oxygen into highly cytotoxic singlet oxygen (^1^O_2_). However, the rigid π-conjugated structure of porphyrins frequently results in the formation of aggregates in aqueous solutions, which leads to the self-quenching of the excited state. Cucurbit[n]urils exhibit the capacity to stably bind with porphyrins via host–guest interactions, effectively inhibiting their aggregation and potentially enhancing the therapeutic efficacy of PDT. In this study, water-soluble tetramethyl cucurbit[6]uril (TMeQ[6]) was selected as the host, while four propionic acid group-appended porphyrin cationic (TPPOR) was utilized as guests to construct a supramolecular photosensitizer (TPPOR-2TMeQ[6]) in a molar ratio of 2:1. Further experimental findings demonstrate that the presence of TMeQ[6] inhibits the aggregation of TPPOR through non-covalent interactions. This inhibition reduces the energy difference between the excited singlet and triplet states, thereby enhancing the conversion efficiency of ^1^O_2_. Moreover, TPPOR-2TMeQ[6] exhibits favorable biocompatibility and minimal dark toxicity against breast cancer cells (4T1). Upon intracellular excitation, the levels of reactive oxygen species (ROS) significantly increase, inducing oxidative stress in 4T1 cells and leading to apoptosis. Consequently, the findings of this study suggest that the enhanced photosensitization achieved through this supramolecular approach is likely to promote the anticancer therapeutic effects of PDT, thereby broadening the application prospects of porphyrins within PDT systems.

## 1. Introduction

In the domain of cancer treatment, photodynamic therapy (PDT) is recognized as a minimally invasive therapeutic approach. The photodynamic effect of PDT can be precisely regulated in proximity to the tumor, thereby minimizing damage to adjacent normal cells and tissues. This technique holds significant potential for enhancing the quality of life and prolonging the survival of patients with advanced cancer [1,2,3]. In PDT, the electrons of the PS are excited from the ground state (S0) to a singlet excited state (Sn) upon exposure to excitation light. During this process, the electrons undergo internal conversion to transition to the lowest energy level of the singlet excited state (S1). Electrons in the S1 state can either induce fluorescence emission and transition back to the ground state via radiative decay or undergo intersystem crossing to enter the excited triplet state (T1). Subsequently, the electrons in the T1 state react with nearby substrates to form radical cations or anions, which can further react with oxygen-containing matrices (such as water and oxygen) to produce reactive oxygen species (ROS), including superoxide anions and hydroxyl radicals. Alternatively, the electrons in the T1 state can directly transfer energy to molecular oxygen, resulting in the generation of highly reactive singlet oxygen (^1^O_2_) [4,5,6,7]. The ROS can inflict damage on cellular membranes, mitochondria, proteins, and DNA, thereby inducing apoptosis and necrosis in cancer cells [8,9]. Porphyrin derivatives (such as dihydroporphyrin, bacteriochlorin, and phthalocyanine) are frequently employed as PS in PDT due to their superior photophysical properties, which include effective absorption in the visible spectrum and prolonged triplet excited states [10,11,12]. However, the π-conjugated structure of porphyrins renders them susceptible to significant intermolecular π–π interactions and hydrophobic effects in aqueous environments, leading to a propensity for aggregation. The aggregation of porphyrins results in self-quenching in the excited state and consequently inhibits the generation of ^1^O_2_ and negatively impacts the effectiveness of PDT [13]. This presents a notable challenge for the utilization of porphyrins in PDT.

To address this issue, prior studies have incorporated hydrophilic substituents with substantial spatial dimensions into the porphyrin structure via organic synthesis strategies to enhance their water solubility and PDT efficiency [14,15]. Porphyrins can be effectively modified by coupling them with amino acids, peptides, and sugars. Furthermore, this modification through coupling can confer targeting capabilities to PS, while these substituents may also function as spacers to prevent the aggregation of porphyrins, thereby improving the therapeutic efficacy of PDT. However, this approach may result in the distortion of the macrocyclic plane of porphyrins, leading to the dissipation of excited state energy through non-radiative transitions. This phenomenon reduces the probability of fluorescence emission and intersystem crossing to the triplet state [16]. Recent research has demonstrated that various organic (such as liposomes, micelles, albumin, and dendrimers) and inorganic (including gold, metal-organic frameworks, and silica) nanocarriers can more effectively disperse porphyrins in aqueous solutions [17,18,19,20] and improve their delivery to tumor sites [21]. Bacteriochlorin, in comparison to other porphyrin derivatives, exhibits its maximum absorption wavelength within the near-infrared region, which corresponds to the transparent window of human tissues in the light spectrum (700 nm–850 nm) and possesses enhanced penetration capabilities [22]. This characteristic effectively mitigates the challenge of inadequate tissue penetration associated with the shorter absorption wavelengths of conventional PS. In the study conducted by Janusz M. Dąbrowski and colleagues, the efficacy of PDT utilizing redaporfin (F2BMet) was significantly improved through the incorporation of Pluronic block copolymers (P123 and F127). The findings indicated that the application of F2BMet-P123 micelles markedly enhanced selectivity, achieving a 100% cure rate and demonstrating long-term efficacy in a mouse melanoma model [23]. Furthermore, these micelles exhibited favorable biocompatibility. Nevertheless, challenges such as premature leakage and the inherent toxicity of the nanocarriers remain substantial obstacles to achieving effective treatment [24,25,26,27]. Consequently, there is an urgent need to develop porphyrin photosensitizers that are characterized by simplicity, high stability, low toxicity, and water solubility.

Cucurbit[n]urils (Q[n]s), classified as fourth-generation supramolecular macrocyclic hosts, feature hydrophobic cavities and carbonyl ports that enable the selective encapsulation of various guest molecules through hydrophobic interactions and ion-dipole interactions [28,29,30]. In comparison to other macrocyclic hosts, such as cyclodextrins and calixarenes, Q[n]s demonstrate a more stable binding affinity with guests in aqueous solutions, particularly for cationic guests. This characteristic contributes to the formation of more stable supramolecular assemblies involving Q[n]s [31,32]. Research indicates that Q[n]s exhibit favorable stability and biocompatibility within biological systems, rendering them suitable candidates for drug delivery applications [33,34]. Importantly, the rigid cavity of Q[n]s can restrict the internal rotation of guest molecules, facilitate electron transfer, and diminish non-radiative transitions. These effects enhance the photophysical properties of the encapsulated guests, thereby leading to the development of light-activated supramolecular therapeutic nano-systems [35,36,37,38,39,40]. For instance, Zhang and colleagues demonstrated that Q[n]s effectively inhibited the aggregation of PS in aqueous solutions, which in turn improved the efficacy of PDT and yielded significant outcomes in tumor treatment and antibacterial applications [41,42,43,44]. Although larger Q[n]s, such as Q[8] or Q[10], exhibit superior capabilities in inhibiting PS aggregation and can even form supramolecular polymers following host–guest interactions, thereby significantly enhancing the production efficiency of ROS [45,46], the limited solubility of Q[8] and Q[10] poses a considerable challenge to the application in PDT.

**Scheme 1:**
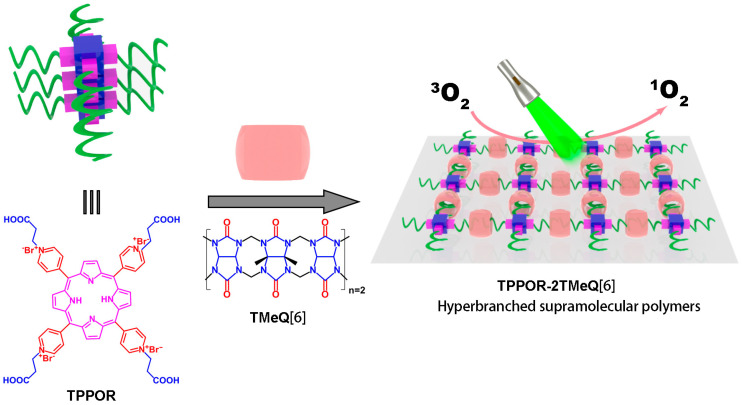
The chemical structures of TPPOR-2TMeQ[6] and a schematic representation of the photosensitizer derived from the host–guest assembly.

In this study, we selected water-soluble tetramethyl cucurbit[6]uril (TMeQ[6]) as the host and four propionic acid group-appended porphyrin cationic (TPPOR) compounds as the guests to design and construct a supramolecular photosensitizer (TPPOR-2TMeQ[6]) (Scheme 1). In comparison to conventional Q[6], TMeQ[6] exhibits not only enhanced water solubility but also, more significantly, an increased spatial size due to the incorporation of four methyl groups into its glycosylurea unit. This larger steric hindrance associated with TMeQ[6] effectively inhibits the aggregation of TPPOR within aqueous solutions. Additionally, the cavity of TMeQ[6] is smaller than those of other Q[n] compounds, such as Q[7], Q[8], or Q[10]. Consequently, it is feasible to encapsulate two carboxyl side chains within the cavity of TMeQ[6], thereby forming a ternary complex that can serve as a foundation for the development of hyperbranched supramolecular polymers. The formation of these supramolecular polymers is anticipated to disrupt the aggregation of TPPOR in aqueous environments, consequently enhancing the efficiency of ^1^O_2_ production. Therefore, TPPOR-2TMeQ[6] exhibits enhanced cytotoxicity and phototoxicity against 4T1 cells, thereby improving the efficacy of PDT. This research seeks to expand the utilization of supramolecular photosensitizers in PDT systems with significant implications for cancer treatment.

## 2. Results and Discussion

**Figure 1:**
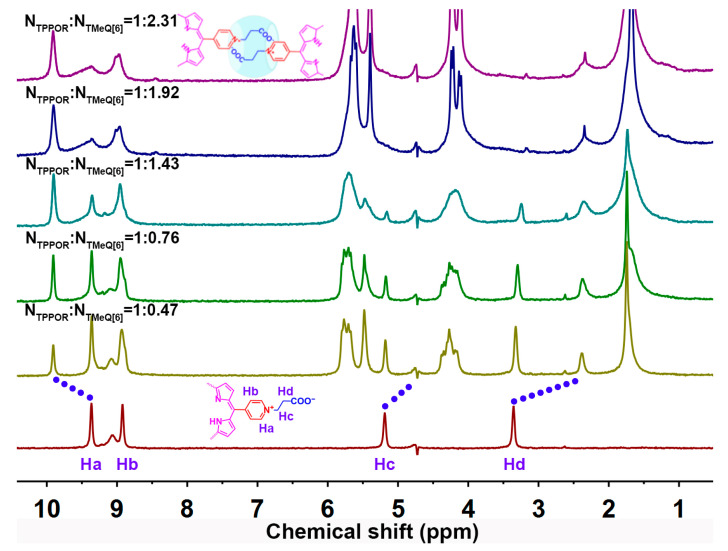
^1^H NMR spectra of TPPOR (1.0 mM) obtained in the presence of varying equivalents of TMeQ[6] in D_2_O.

The TPPOR guests were synthesized through the reaction of 5,10,15,20-Tetra(4-pyridyl)porphyrin with 3-bromopropionic acid, resulting in a yield of 68.73%. The synthesized products were characterized using ^1^H nuclear magnetic resonance (NMR) and mass spectrometry (MS), as illustrated in Figures S1 and S2. The molecular binding behavior of TPPOR with TMeQ[6] was investigated utilizing ^1^H NMR titration. The concentration of TPPOR was kept constant while varying molar concentrations of deuterated water solutions of TMeQ[6] were incrementally added. As presented in Figure 1, the chemical shifts of the methylene protons (Hc, Hd) on the carboxyl side chain of the guest TPPOR towards higher magnetic fields upon the addition of TMeQ[6] can be attributed to the shielding effect exerted by the host. This phenomenon suggests that the carboxyl side chain of the guest TPPOR is encapsulated within the cavity of TMeQ[6]. Conversely, the chemical shift of the α proton (Ha) on the pyridine ring of TPPOR shifts to lower magnetic fields, indicating its location at the port of TMeQ[6], where it is affected by the deshielding effect of cucurbituril. Notably, no change was observed in the chemical shift of the b protons on the pyridine ring, indicating that TMeQ[6] exclusively encapsulated the carboxyl side chain of TPPOR. Furthermore, at the onset of the titration, the methylene proton peaks on TMeQ[6] displayed splitting; as the molar ratio of host to guest approached 2:1, these peaks gradually returned, signifying that one carboxyl side chain entered each port of TMeQ[6], resulting in a relatively symmetrical environment for the host. Additional thermodynamic insights regarding the host–guest binding were obtained through isothermal titration calorimetry (ITC) (Figure S3), which indicated a stoichiometric ratio of 1:2 for the TPPOR and TMeQ[6] complex, with a binding constant of 6.02 × 10^4^ M^−1^. Both the changes in enthalpy (ΔH) and entropy (ΔS) were found to be negative, suggesting that the interaction between TPPOR and TMeQ[6] is a spontaneous process driven by both enthalpic and entropic contributions. Given the host–guest ratio of 2:1 and the encapsulation of two carboxyl side chains within the cavity of TMeQ[6], this binding may facilitate the formation of TPPOR-2TMeQ[6].

**Figure 2:**
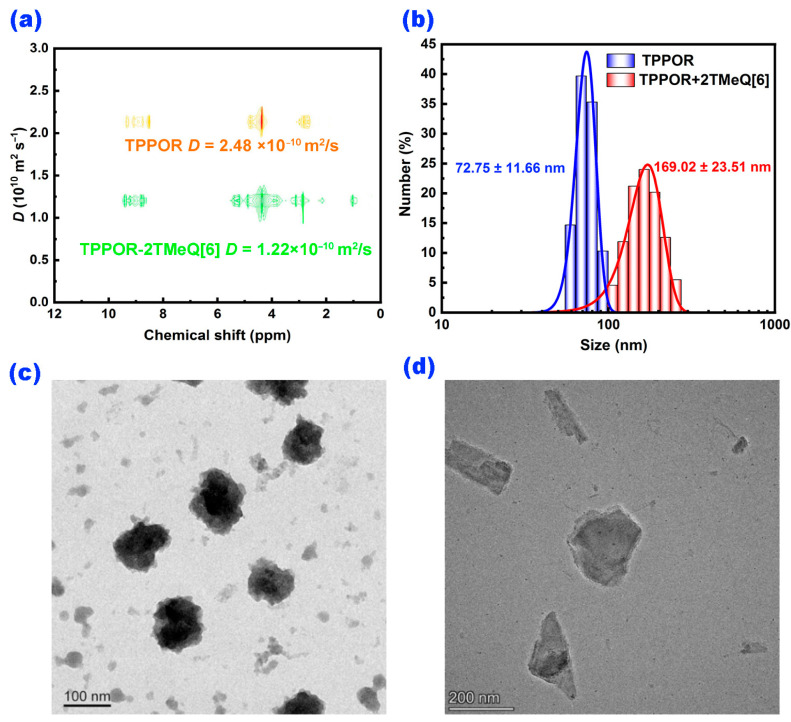
(**a**) DOSY spectra and (**b**) DLS results for TPPOR and TPPOR-2TMeQ[6]; TEM images of (**c**) TPPOR and (**d**) TPPOR-2TMeQ[6] in aqueous solution.

Diffusion-ordered nuclear magnetic resonance (DOSY) spectroscopy, which facilitates the determination of the diffusion coefficient of samples, was employed to further validate the formation of TPPOR-2TMeQ[6]. As depicted in Figure 2a, the diffusion coefficient of TPPOR was measured at 2.48 × 10^−10^ m^2^/s. In contrast, the NMR signals of TPPOR-2TMeQ[6] exhibited a singular diffusion coefficient with a reduced value of D = 1.22 × 10^−10^ m^2^/s. This reduction in the diffusion coefficient suggests the formation of larger supramolecular polymers through self-assembly. Dynamic light scattering (DLS) analysis corroborates these findings. As illustrated in Figure 2b, the amphiphilic characteristics of TPPOR molecules enable them to self-assemble into aggregates in aqueous environments, yielding an average hydrated diameter of approximately 70 nm. However, upon the addition of twice the amount of TMeQ[6], the self-assembled structures of TPPOR were no longer detectable, and larger aggregates with a hydrated diameter of approximately 200 nm were observed. This indicates that the self-assembly of TPPOR-2TMeQ[6] significantly differs from that of TPPOR. Moreover, electron microscopy observations revealed alterations in the morphology of the self-assembled structures following the addition of twice the amount of TMeQ[6] to the aqueous solution of TPPOR. As shown in Figure 2c and Figure S4, TPPOR self-assembled into spherical-like aggregates, exhibiting no significant distinction between the center and the periphery of the spheres, which suggests a micellar structure. Conversely, transmission electron microscopy (TEM) and atomic force microscopy (AFM) images (Figure 2d and Figure S5) indicate that TPPOR-2TMeQ[6] self-assembled into nanosheets, resembling the expanded structure hypothesized from the connection of two TPPOR molecules via TMeQ[6]. This evidence further substantiates the formation of hyperbranched supramolecular polymers, TPPOR-2TMeQ[6].

**Figure 3:**
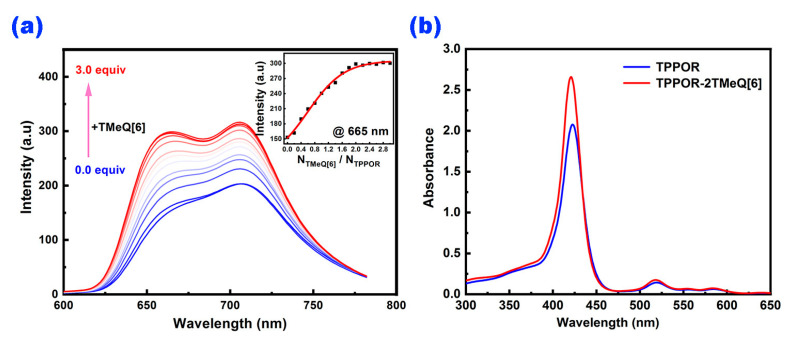
(**a**) FL spectra for TPPOR (20 μM) in an aqueous solution (pH 7.2) at 298 K, with increasing concentrations of the TMeQ[6] host (λ_ex_ = 426 nm). The inset illustrates the change in intensity at 665 nm as a function of the ratio NTMeQ[6]/NTPPOR. (**b**) UV-Vis spectra of TPPOR and the TPPOR-2TMeQ[6] complex with a concentration of TPPOR at 20 μM and pH = 7.2.

The formation of supramolecular polymers resulted in a notable increase in the fluorescence (FL) intensity of TPPOR. As illustrated in Figure 3a, the FL spectra of TPPOR displayed characteristic self-quenching behavior typical of porphyrins, spanning a broad wavelength range from 640 nm to 760 nm. This phenomenon can be attributed to the aggregation-induced FL quenching of π-conjugated chromophores in aqueous solutions. Upon the introduction of the monomer TMeQ[6] hosts into the aqueous environment, the resulting TPPOR-2TMeQ[6] solution exhibited a significantly enhanced FL, with the FL quantum yield rising from 0.75% to 3.77% (Figure S6). This enhancement is due to the formation of supramolecular polymers, wherein four bulky TMeQ[6] hosts encircle the TPPOR guests, thereby inhibiting the aggregation of TPPOR through non-covalent interactions. Furthermore, the photostability of the TPPOR-2TMeQ[6]-based host–guest interaction was assessed in PBS and a 5% DMSO solution. The minimal changes in fluorescence suggest that TPPOR-2TMeQ[6] exhibits commendable photostability in these environments, and the TPPOR-2TMeQ[6] supramolecular polymers were able to maintain stable formation across various solutions (Figure S7). In addition, as depicted in Figure 3b, the molar extinction coefficient of the porphyrin increases upon the addition of TMeQ[6] to the TPPOR aqueous solution, particularly demonstrating a substantial increase in absorption at 423 nm, which corresponds to a prominent Soret band. Given that the molar extinction coefficient of the chromophore is contingent upon the distance between adjacent chromophores [35], the observed increase in porphyrin absorption suggests an increase in the inter-porphyrin distance. Consequently, the formation of supramolecular polymers diminishes the interactions between the chromophores, thereby suppressing the self-quenching of the excited state and restoring the fluorescence of the TPPOR guests.

**Figure 4:**
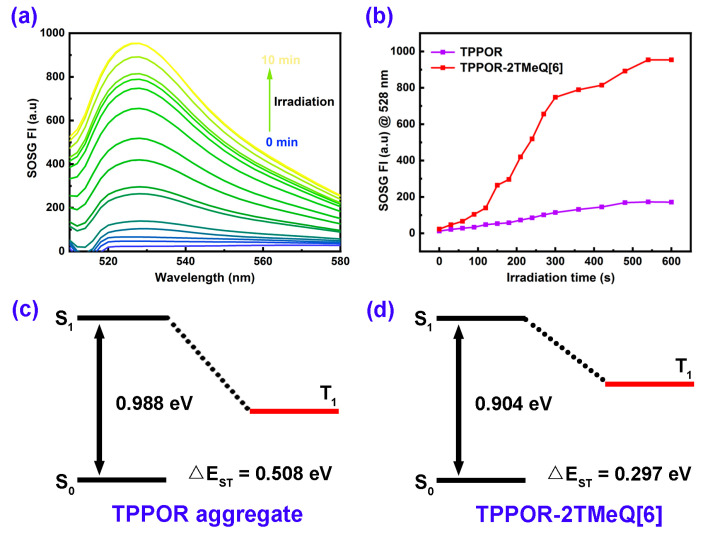
(**a**) FL intensity of SOSG (5.0 μM) in the presence of TPPOR-2TMeQ[6] (20 μM, pH = 7.2), (**b**) the absorbance of SOSG as a function of irradiation time, ΔE_ST_ of (**c**) TPPOR and (**d**) TPPOR-2TMeQ[6].

Next, we assess the TPPOR-2TMeQ[6] supramolecular polymers for their ability to ^1^O_2_, with the aim of evaluating their potential as PS in PDT. As shown in Figure 4a, the commercial Singlet Oxygen Sensor Green (SOSG) was employed to quantify the relative amount of ^1^O_2_ produced. The product resulting from the reaction between ^1^O_2_ and SOSG displays a maximum emission wavelength of 525 nm and exhibits green fluorescence [47,48,49]. The concentration of TPPOR guests was maintained at 20 μM, and under irradiation with a 520 nm laser (500 mW/cm^2^), the amount of ^1^O_2_ generated by TPPOR-2TMeQ[6] was approximately five times greater than that produced by TPPOR, as indicated by the FL changes observed in SOSG (Figure 4b). Due to the close aggregation of porphyrins in solution, these compounds tend to dissipate energy through non-radiative transitions (such as heat) following excitation by visible light, which suppresses the inter-system relaxation process and adversely affects the conversion of ^1^O_2_. However, the formation of TPPOR-2TMeQ[6] supramolecular polymers mitigated the aggregation of TPPOR through host–guest interactions, thereby enhancing the efficiency of the PS in generating ^1^O_2_. Furthermore, the conversion of the PS from the singlet state (S1) to the triplet state (T1) via inter-system relaxation is a critical step in the photosensitization process, directly influencing the conversion efficiency of ^1^O_2_ [39,40]. The energy of the band between S1 and T1 (ΔE_ST_) for TPPOR and TPPOR-2TMeQ[6] was calculated (Figure 4c,d). Notably, in comparison to aggregated TPPOR (ΔE_ST_ = 0.508 eV), TPPOR-2TMeQ[6] exhibited a ΔE_ST_ of 0.297 eV. The formation of TPPOR-2TMeQ[6] supramolecular polymers inhibited the aggregation of TPPOR, thereby facilitating the inter-system relaxation process. This resulted in a significant reduction in the energy difference between the S1 and T1, consequently promoting a more efficient generation of ROS.

**Figure 5:**
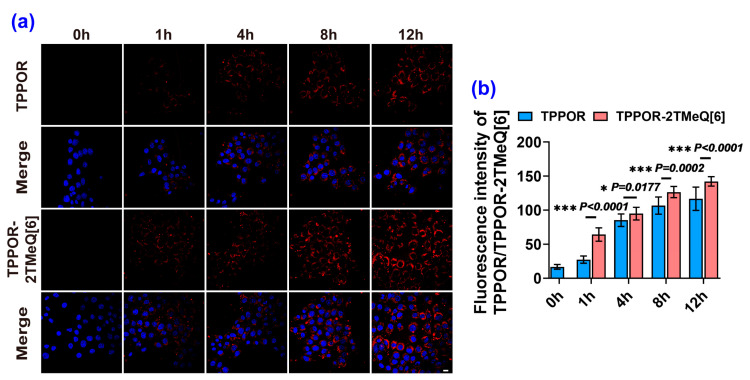
(**a**) TPPOR and TPPOR-2TMeQ[6] cellular uptake in 4T1 cells. Scale bar: 10 μM. (**b**) Quantification of FL intensity (*n* = 12). Data are presented as mean ± standard deviation (SD), with statistical significance at *p* < 0.05.

The internalization of TPPOR-2TMeQ[6] within tumor cells is essential for its effective anti-tumor activity. Consequently, laser confocal scanning microscopy (CLSM) was employed to investigate the uptake of TPPOR-2TMeQ[6] by 4T1 cells. As displayed in Figure 5a,b, under excitation at 561 nm in CLSM, TPPOR, and TPPOR-2TMeQ[6] exhibited red fluorescence. The free TPPOR demonstrated red fluorescence after 4 h of incubation, whereas TPPOR-2TMeQ[6] displayed red fluorescence after only 1 h of incubation. This observation suggests that the stable binding of TPPOR to TMe[6] enhances the FL intensity of TPPOR, corroborating previous findings regarding FL enhancement (Figure 3a). It is essential to recognize that the differing sizes of TPPOR and TPPOR-2TMeQ may result in distinct cellular uptake pathways. It is essential to recognize that the differing sizes and shapes between TPPOR and TPPOR-2TMeQ may result in distinct cellular uptake pathways [50,51]. Consequently, the observed differences in their fluorescence intensity may also be attributed to the varying levels of cellular uptake. Furthermore, as the incubation time increased, the red fluorescence progressively intensified, indicating the efficient internalization of TPPOR-2TMeQ[6] in 4T1 cells, which is advantageous for subsequent photodynamic therapy.

**Figure 6:**
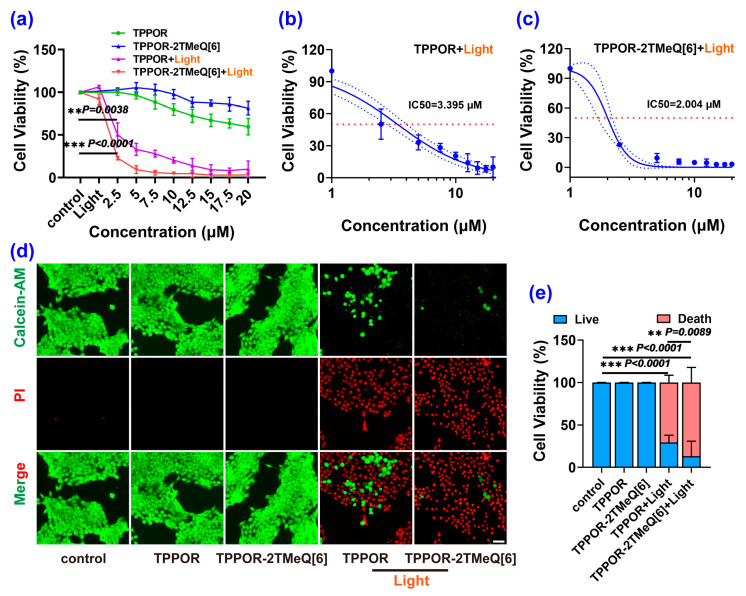
(**a**) The cell viability of 4T1 cells after treatment with different doses of TPPOR and TPPOR-2TMeQ[6] under 520 nm laser irradiation for 3 min (*n* = 3). (**b**) The IC50 of 4T1 cells treated with different doses of TPPOR and lighting (*n* = 3). (**c**) The IC50 of 4T1 cells treated with different doses of TPPOR-2TMeQ[6] and lighting (*n* = 3). (**d**) Calcein AM/PI double staining of live/dead cells in 4T1 cells after drug intervention, scale bar = 50 μm, and (**e**) corresponding quantification of FL intensity (*n* = 12). Data are presented as mean ± SD, with statistical significance at *p* < 0.05.

Considering that TPPOR can produce ^1^O_2_ under 520 nm laser irradiation, which has a toxic effect on cells [52], we therefore conducted in vitro phototoxicity assessments. The liver serves as the primary organ for drug metabolism; therefore, we selected normal human liver cells (LO2) as a cellular model to assess the safety of the drugs under investigation. Utilizing the Cell Counting Kit-8 (CCK-8) method, we investigated the effects of free TPPOR and TPPOR-2TMeQ[6] on the viability of LO2 cells, both with and without exposure to 520 nm laser irradiation (500 mW/cm^2^, 3 min). As illustrated in Figure S8, in the absence of laser irradiation, the free TPPOR group exhibited approximately 50% cell survival at a maximum concentration of 20 μM, indicating cytotoxicity at this concentration. Conversely, the TPPOR-2TMeQ[6] group demonstrated nearly 80% cell survival, suggesting that TPPOR-2TMeQ[6] supramolecular polymers possess favorable cell compatibility, which may mitigate the toxic side effects of the drug during systemic circulation. Under 520 nm laser irradiation, both TPPOR and TPPOR-2TMeQ[6] exhibited a dose-dependent reduction in LO2 cell survival as the concentration increased (Figure S8), thereby confirming their phototoxic effects on LO2 cells. This finding implies that the application of laser irradiation to tumor sites may inadvertently damage normal cells within the tumor vicinity, presenting challenges for future research. Subsequently, we evaluated the cytotoxic effects of the drugs on 4T1 cells, as illustrated in Figure 6. Due to the stable binding of TMeQ[6] with TPPOR, it exhibited favorable cellular safety for 4T1 cells within the concentration range of 0–20 μM in the absence of light irradiation. Following light exposure, it also demonstrated dose-dependent cytotoxicity against 4T1 cells.

The IC50 value for the free TPPOR group was determined to be 3.395 μM (Figure 6b), while the IC50 for the TPPOR-2TMeQ[6] group was 2.004 μM (Figure 6c), indicating that TPPOR-2TMeQ[6] demonstrates enhanced phototoxicity. Furthermore, to visually evaluate the phototoxic effects of TPPOR-2TMeQ[6], we treated 4T1 cells with a concentration of 5 μM and subsequently incubated the cells with the Calcein AM/PI live/dead cell dual staining probe (Figure 6d,e). Calcein AM stains viable cells, emitting green fluorescence, while propidium iodide (PI) stains non-viable cells, emitting red fluorescence. The control group, along with the TPPOR and TPPOR-2TMeQ[6] treatment groups, exhibited strong green fluorescence with minimal red fluorescence, indicating that none of these treatments induced cell death in 4T1 cells. In contrast, the TPPOR + Light and TPPOR-2TMeQ[6] + Light groups displayed red fluorescence signals, signifying that 520 nm laser irradiation of TPPOR can induce cell death in 4T1 tumor cells, with the TPPOR-2TMeQ[6] + Light group exhibiting more pronounced red fluorescence and a stronger cytotoxic effect. Therefore, the stable encapsulation of TPPOR by TMeQ[6] allows TPPOR-2TMeQ[6] supramolecular polymers to demonstrate good cell compatibility while effectively inducing cell death in 4T1 cells following 520 nm laser irradiation.

**Figure 7:**
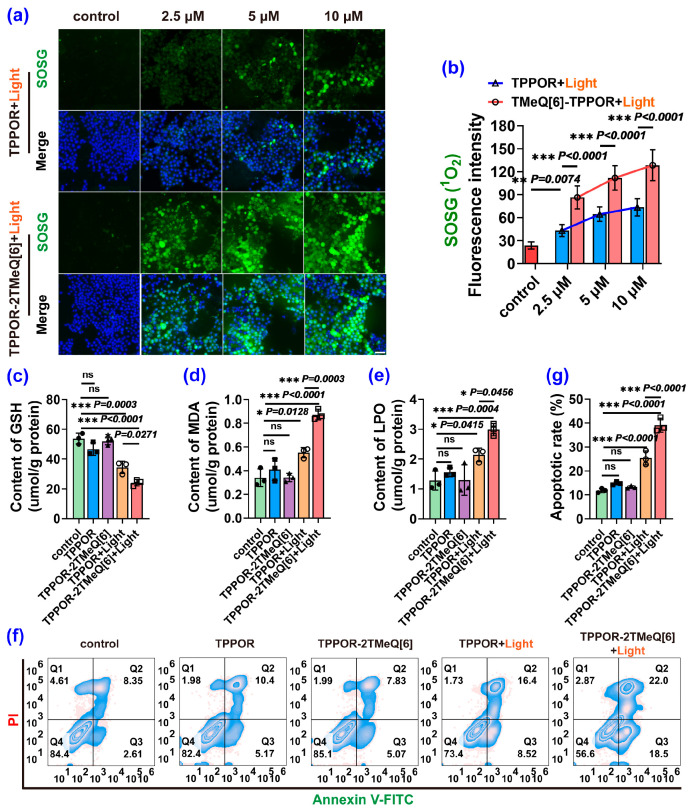
(**a**) The levels of intracellular ^1^O_2_ in 4T1 cells after treatment with different doses of TPPOR and TPPOR-2TMeQ[6] under 520 nm laser irradiation for 3 min, scale bar = 50 μm, (**b**) corresponding quantification of ^1^O_2_ (*n* = 12). The content of (**c**) GSH, (**d**) MDA, and (**e**) LPO in 4T1 cells after drug intervention (*n* = 3). (**f**) Flow cytometry analysis of apoptosis in 4T1 cells after drug intervention, and (**g**) corresponding statistical analysis of apoptosis (early + late) results (*n* = 3). Data are presented as mean ± SD, with statistical significance at *p* < 0.05.

^1^O_2_ is a type of ROS [53], and the production of intracellular ROS is crucial for the phototoxicity of TPPOR-2TMeQ[6] under light exposure. First, we assessed the capacity of TPPOR-2TMeQ[6] to generate ^1^O_2_ in 4T1 cells utilizing the SOSG probe [48,49]. The results from the inverted fluorescence microscope (IFM) showed that, compared to the control group, there was green fluorescence in the cells of both the TPPOR + Light and TPPOR-2TMeQ[6] + Light groups at different concentrations (Figure 7a,b), and this fluorescence exhibited a concentration-dependent manner, indicating that ^1^O_2_ was produced in all groups of 4T1 cells. Notably, the green fluorescence in the TPPOR-2TMeQ[6] + Light group was significantly stronger than that in the TPPOR + Light group, suggesting that the ROS levels were higher in the TPPOR-2TMeQ[6] + Light group. This enhancement is attributed to the encapsulation of TPPOR by TMe[6], which inhibits its aggregation and consequently augments the ability of TPPOR-2TMeQ[6] to generate ^1^O_2_ upon light exposure. We further employed 2,7-dichlorodihydrofluorescein diacetate (DCFH-DA) as a probe to evaluate the impact of varying doses of TPPOR-2TMeQ[6] on ROS generation in 4T1 cells following 3 min of irradiation with a 520 nm laser. The findings are in alignment with the results of the SOSG study (Figure S9a,b), demonstrating that ROS was produced in all groups of 4T1 cells, with the highest levels observed in the TPPOR-2TMeQ[6] + Light group, attributable to its superior capacity for ^1^O_2_ production.

The accumulation of intracellular ROS can trigger lipid peroxidation (LPO), during which free radical scavengers such as GSH are consumed, leading to the production of malondialdehyde (MDA) and other products that induce oxidative stress (OS) in cells [53]. Therefore, we used a corresponding kit to detect changes in GSH, MDA, and LPO levels in the 4T1 cells of each group to assess the degree of OS. The results are shown in Figure 7c–e, indicating that there were no statistically significant differences in the concentrations of GSH, MDA, and LPO among the control group, TPPOR, and TPPOR-2TMeQ[6] treatment groups, suggesting that TPPOR and TPPOR-2TMeQ[6] at a concentration of 5 μM did not induce OS in 4T1 cells. However, in the TPPOR + Light and TPPOR-2TMeQ[6] + Light treatment groups, GSH levels reduced while MDA and LPO levels increased, indicating that OS occurred intracellularly. The effect was more pronounced in the TPPOR-2TMeQ[6] + Light treatment group, suggesting that lipid peroxidation was more severe in this group, making it easier to induce OS and subsequently lead to cell apoptosis. To verify this conclusion, we assessed the apoptosis of cells in each group using the FITC-Annexin V and PI double staining method along with flow cytometry. The results showed (Figure 7f,g) that the proportion of apoptotic cells in the TPPOR-2TMeQ[6] + Light group (approximately 40.5%) was higher than that in the TPPOR + Light group (approximately 24.92%). Based on the findings presented above, it can be concluded that TMeQ[6] and TPPOR exhibit stable binding through host–guest interactions to form TPPOR-2TMeQ[6] supramolecular polymers. This interaction effectively inhibits the aggregation of TPPOR and enhances its capacity to generate ^1^O_2_ upon exposure to 520 nm laser irradiation, ultimately leading to the induction of apoptosis in 4T1 cells.

## 3. Materials and Methods

### 3.1. Materials

All materials listed below were of analytical purity grade and were utilized without further purification. We procured 5,10,15,20-Tetra(4-pyridyl)porphyrin from an unspecified source. 3-Bromopropionic acid was obtained from TCI. TMeQ[6] was provided by the Key Laboratory of Macrocyclic and Supramolecular Chemistry of Guizhou Province. Dulbecco’s Modified Eagle Medium (DMEM) and penicillin–streptomycin were purchased from Gibco (Waltham, MA, USA). Fetal bovine serum (FBS) was acquired from Umedium (Hefei, China). Phosphate-buffered saline (PBS) and phenylmethanesulfonyl fluoride (PMSF) were obtained from Solarbio (Beijing, China). Hoechst 33342, CCK-8, Calcein-AM/PI live/dead cell staining kit, SOSG, DCFH-DA, and RIPA protein lysate were purchased from Beyotime (Shanghai, China). Glutathione (GSH), malondialdehyde (MDA) and lipid peroxidation (LPO) assay kits were obtained from Jiancheng (Nanjing, China). The apoptosis assay kit was acquired from KeyGEN (Nanjing, China).

### 3.2. Instruments

A JEOL JNM-ECZ400S nuclear magnetic resonance spectrometer (JEOL, Tokyo, Japan) was utilized to acquire ^1^H NMR spectra (Bruker, Billerica, MA, USA). Ultraviolet–visible (UV-Vis) spectroscopy data were collected using a SHIMADZU UV-2600i spectrophotometer (Shimadzu, Kyoto, Japan). Transmission electron microscopy (TEM) images were obtained with an FET Talors F200C field emission transmission electron microscope (Thermo Fisher Scientific, Waltham, MA, USA). Isothermal titration calorimetry (ITC) experiments were conducted using a NANO ITC (TA, New Castle, DE, USA). Dynamic light scattering (DLS) measurements were performed with a NanoBrook 90Plus (Brookhaven, New York, NY, USA). FL quantum yields were assessed in aqueous solution (20 μM, pH 7.2) using an FLS1000 Photoluminescence Spectrometer (Edinburgh, Livingston, UK). This had an excitation range from 434 to 452 nm, and a luminescence range from 649.00 to 758.00 nm. The laser confocal microscope employed was an Olympus Spin 10 (Olympus, Tokyo, Japan). Inverted fluorescence microscopy was conducted using instruments from TI-SR (Nikon, Tokyo, Japan). The lossy cell analyzer utilized was a BD FACSCelesta (BD, Franklin Lakes, NJ, USA). The microplate reader used in the experiments was an Allsheng model (Allsheng, Hangzhou, China).

### 3.3. Synthesis of TPPOR

We dissolved 5,10,15,20-Tetra(4-pyridyl)porphyrin (310 mg, 0.5 mmol) and 3-bromopropionic acid (610 mg, 4 mmol) in 10 mL dimethylformamide (DMF). The resulting solution was stirred at 120 °C for 8 h under a nitrogen atmosphere. Following this period, the solution was allowed to cool and subsequently filtered. The solid precipitate was thoroughly washed with trichloromethane and diethyl ether, and then dried under vacuum conditions. The overall yield of the reaction was determined to be 58%.

### 3.4. ^1^H NMR Measurements

The ^1^H NMR spectra were recorded at a temperature of 25 °C. Deuterium oxide (D_2_O) was employed for field frequency locking, and the observed chemical shifts were reported in parts per million relative to the internal standard tetramethylsilane (TMS), which is assigned a value of 0 ppm for ^1^H. ^1^H NMR titrations were conducted using solutions that maintained a constant concentration of TPPOR (5.00 mM, 500 μL) but varying concentrations of TMeQ[6].

### 3.5. Isothermal Titration Calorimetry (ITC)

All sample solutions for titration were prepared in a 5 mM phosphate buffer with a pH of 7.2. In a typical titration experiment, TPPOR was contained in the injection syringe at a concentration of 0.5 mM, while TMeQ[6] was present in the sample cell at a concentration starting from 0.05 mM. Each titration protocol included an initial injection of 0.4 μL to eliminate preliminary errors. This was followed by 50 consecutive injections of 1 μL, with a 180-s interval between each injection. Prior to titration, all solutions were degassed. The temperature during the titration was maintained at 37 °C. The isothermal titration calorimetry (ITC) curves were analyzed using Origin 7, employing a one-site binding model to determine the macroscopic changes in enthalpy, entropy, and the association constant.

### 3.6. Singlet Oxygen Generation Experiments

The concentration of singlet oxygen generated in the solution was assessed. A 5 mM solution of SOSG was prepared by dissolving it in methanol, and this solution was freshly prepared at the beginning of each experiment. TPPOR and TPPOR-2TMeQ (with a concentration of TPPOR = 20.0 µM) were combined with SOSG (5 µM) in a 5 mM sodium phosphate buffer at pH 7.2. The samples were irradiated using a laser with an intensity of 500 mW/cm^2^ at a wavelength of 520 nm. The fluorescence of SOSG was measured at 30-s intervals by exciting the samples at 495 nm and collecting emissions between 505 and 650 nm.

### 3.7. DFT Calculations

The DFT calculations are performed via the ORCA program (Version 5.0) [54,55] and the quick input is powered by Multiwfn (Version 3.0) [56]. The geometry of the studied system was optimized based on the B97-3c method [57]. All structures are visualized using GaussView (Version 5.0). The spin–orbit coupling matrix element is calculated based on the TDDFT.

### 3.8. Cell Culture and Cell Experiment

The 4T1 cell line was obtained from GAINING in Shanghai, China (Catalog No. CM-M029). LO2 cell lines were held and cultured in the laboratory Molecular Medicine Engineering Research Center, Guizhou Medical University. The cells were cultured in high-glucose DMEM supplemented with 1% penicillin/streptomycin and 10% fetal bovine serum (FBS). The cultures were maintained in a humidified incubator at 37 °C with an atmosphere of 5% CO_2_.

### 3.9. Cellular Uptake

The 4T1 cells were seeded into a laser confocal cell culture dish. Following a 24-h incubation period, the culture medium was replaced with DMEM containing TPPOR and TPPOR-2TMeQ[6] at a concentration of 20 µM. Subsequently, the cells were incubated with TPPOR and TPPOR-2TMeQ[6] for varying durations. Cell fluorescence imaging was conducted using a confocal laser scanning microscope (CLSM) with an excitation wavelength of 561 nm.

### 3.10. Cytotoxicity Test

Cell viability was measured using CCK-8. After an incubation period in a 96-well culture plate containing 8 × 10^3^ cells per well and 100 μL of growth media, the media were aspirated. The cells were then treated with varying concentrations of TPPOR and TPPOR-2TMeQ[6] (0, 2.5, 5, 7.5, 10, 12.5, 15, 17.5, and 20 μM), which were diluted in the prepared medium. Notably, the light treatment groups were exposed to a 520 nm laser for 3 min at an intensity of 500 mW/cm^2^ after a 4-h incubation period with the drug. Subsequently, each well was incubated with 10 μL of CCK-8 solution in the cell incubator for 1 h, after which the absorbance was measured at 450 nm using a microplate reader.

### 3.11. Calcein-AM/PI Live/Dead Cell Dual Staining

Cell viability was evaluated using the Cell Counting Kit-8 (CCK-8) assay. Cells were seeded on a 24-well plate and incubated overnight. Subsequently, we added 5 μM of TPPOR and TPPOR-2TMeQ[6] to the cells and incubated them for 4 h. Following this incubation, the light groups were irradiated with a 520 nm laser for 3 min at an intensity of 500 mW/cm^2^. After 24 h, each well was washed three times with PBS. Each well was then incubated with 250 μL of DMEM, 2 μL of Calcein-AM, and 4 μL of propidium iodide (PI) for 30 min in the incubator. Finally, each well was washed three times with PBS and observed using an inverted fluorescence microscope (Nikon, Tokyo, Japan).

### 3.12. ^1^O_2_ Assay

Cells were seeded in 24-well plates and incubated overnight in a cell incubator. Various concentrations of TPPOR and TPPOR-2TMeQ[6] were then incubated with the cells and subsequently irradiated with a 520 nm laser for 3 min at an intensity of 500 mW/cm^2^ after a 4-h incubation period. Following a 16-h incubation, each well was washed three times with phosphate-buffered saline (PBS), and 1 mL of 10 μM SOSG in DMEM was added. The plates were then placed back in the cell incubator for 60 min. After this incubation, the cells were incubated with Hoechst 33342. At last, the cells were washed three times with PBS and 1 mL of DMEM was added. Finally, the cells were observed using an inverted fluorescence microscope.

### 3.13. ROS Assay

Cells were seeded in 24-well plates and incubated overnight in a cell incubator. Various concentrations of TPPOR and TPPOR-2TMeQ[6] were then incubated with the cells and subsequently irradiated with a 520 nm laser for 3 min at an intensity of 500 mW/cm^2^ after a 4-h incubation period. Following 16-h incubation, each well was washed three times with phosphate-buffered saline (PBS), and 1 mL of 15 μM DCFH-DA in DMEM was added. The plates were then placed back in the cell incubator for 30 min. After incubation, the cells were washed three times with PBS and 1 mL of DMEM was added. Finally, the cells were observed using an inverted fluorescence microscope.

### 3.14. Detection of GSH, Reduced MDA and LPO

Cells were cultured in 100 mm dishes within a cell incubator overnight. Subsequently, we introduced 5 μM of TPPOR and TPPOR-2TMeQ[6] to the cells. After a 4-h incubation period, the light groups were irradiated with a 520 nm laser for 10 min at an intensity of 500 mW/cm^2^. Following a 24-h incubation period, the cells were harvested via centrifugation at 2000 rpm for 5 min and washed three times with PBS. Subsequently, 300 μL of RIPA protein lysis buffer (containing 1% PMSF) was added to each tube. The cells were then lysed using an ultrasonic homogenizer set to 120 W. This operated for 10 s, and was paused for 10 s. We repeated this cycle three times. Afterward, the lysates were placed on ice and allowed to continue lysing for an additional 30 min. Finally, the proteins were centrifuged at 12,000 rpm for 25 min, and the supernatant was analyzed according to the manufacturer’s instructions. The absorbance of each well was measured using a microplate reader.

### 3.15. Annexin V-FITC/PI Apoptosis Assay

Cells were cultured in 60 mm dishes within a cell incubator overnight. Subsequently, 5 μM of TPPOR and TPPOR-2TMeQ[6] were introduced to the cells. After a 4-h incubation period, the light groups were irradiated with a 520 nm laser for 5 min at an intensity of 500 mW/cm^2^. Following a 24-h incubation, the cells were harvested by centrifugation at 2000 rpm for 5 min and washed three times with PBS. Each tube was then supplemented with 500 μL of staining buffer, along with 5 μL of fluorescein isothiocyanate (FITC) and 5 μL of propidium iodide (PI), with the exception of the single-stained and blank tubes, which were allowed to stand at room temperature for 15 min. Finally, fluorescence intensity was measured using flow cytometry.

### 3.16. Statistical Analysis

The experimental results are expressed as mean ± standard deviation (SD). The experiments were conducted in triplicate, comprising three independent trials. A Student’s *t*-test was utilized to evaluate the statistical significance of differences between two data groups, whereas one-way ANOVA was employed to assess differences among multiple data groups. Data processing and image generation were performed using GraphPad Prism (version 8.0.2) and Origin 2021 software, with a *p*-value of less than 0.05 indicating statistically significant differences.

## 4. Conclusions

In summary, the water-soluble TMeQ[6] was selected as the host for the construction of the supramolecular photosensitizer TPPOR-2TMeQ[6] in conjunction with TPPOR. ^1^H NMR titration confirmed that the carboxyl side chain of TPPOR is encapsulated within the cavity of TMeQ[6], with the molar ratio of hosts to guests approaching 2:1. ITC results indicated that the binding constant between TPPOR and TMeQ[6] was 6.02 × 10^4^ M^−1^. Additionally, DOSY, DLS, and TEM results further validated the formation of TPPOR-2TMeQ[6], which resulted in a significant increase in the FL intensity of TPPOR. The ΔE_ST_ of 0.297 eV for TPPOR-2TMeQ[6] was notably reduced compared to that of TPPOR (0.508 eV), indicating that the formation of TPPOR-2TMeQ[6] mitigated the aggregation of TPPOR through host–guest interactions, thereby enhancing the efficiency of ^1^O_2_ generation. In the absence of laser irradiation, the free TPPOR group exhibited approximately 60% cell survival, while the TPPOR-2TMeQ[6] group demonstrated nearly 80% cell survival, suggesting that TPPOR-2TMeQ[6] possesses favorable cell compatibility. Under 520 nm laser irradiation, the IC50 value for the free TPPOR group was determined to be 3.395 μM, whereas the IC50 for the TPPOR-2TMeQ[6] group was 2.004 μM, indicating that TPPOR-2TMeQ[6] exhibits enhanced phototoxicity. The DCFH-DA probe was employed to assess ROS generation in 4T1 cells induced by TPPOR-2TMeQ[6]. The results suggested that the encapsulation of TPPOR by TMeQ[6] can enhance its ability to generate ^1^O_2_ under light exposure. Analyses of GSH, MDA, and LPO indicated that TPPOR-2TMeQ[6] can significantly induce OS in 4T1 tumor cells, ultimately leading to cell apoptosis. This study is a preliminary investigation into the application of TPPOR in PDT. The 520 nm green laser utilized during the treatment demonstrates limited penetration capabilities, making TPPOR-2TMeQ[6] particularly suitable for targeting superficial tumors, such as skin cancer and melanoma. Future advancements should focus on replacing porphyrin with bacteriochlorin in the construction of a supramolecular photosensitizer, aimed at improving absorption in the near-infrared spectrum.

## Data Availability

The original data are available from the corresponding author upon reasonable request.

## Supplementary Materials

The following supporting information can be downloaded at: https://www.mdpi.com/article/10.3390/ijms252313037/s1.
