# Supplementary material for: Tetramethyl Cucurbit[6]uril–Porphyrin Supramolecular Polymer Enhances Photosensitization
Source: Int J Mol Sci. 2024 Dec 4;25(23):13037. doi: 10.3390/ijms252313037 (PMC11641528; doi:10.3390/ijms252313037)
Supplement: Supplementary file 1 [file ijms-25-13037-s001.zip › ijms-3324809-supplementary.pdf]

Supplementary Information

Tetramethyl Cucurbit[6]uril–Porphyrin Supramolecular Polymer Enhances Photosensitization

Bo Xiao[†], Yueyue Liao[†], Jinyu Zhang, Ke Chen, Guangwei Feng, Jian Feng* and Chunlin Zhang*

School of Basic Medical Sciences/ School of Medical Humanities, Guizhou Medical University, Guiyang 550025, China

* Corresponding authors.

E-mail address: jfeng@gmc.edu.cn (Jian Feng); chunlinzhang@gmc.edu.cn (Chunlin Zhang)

[†] These authors contributed equally to this work.

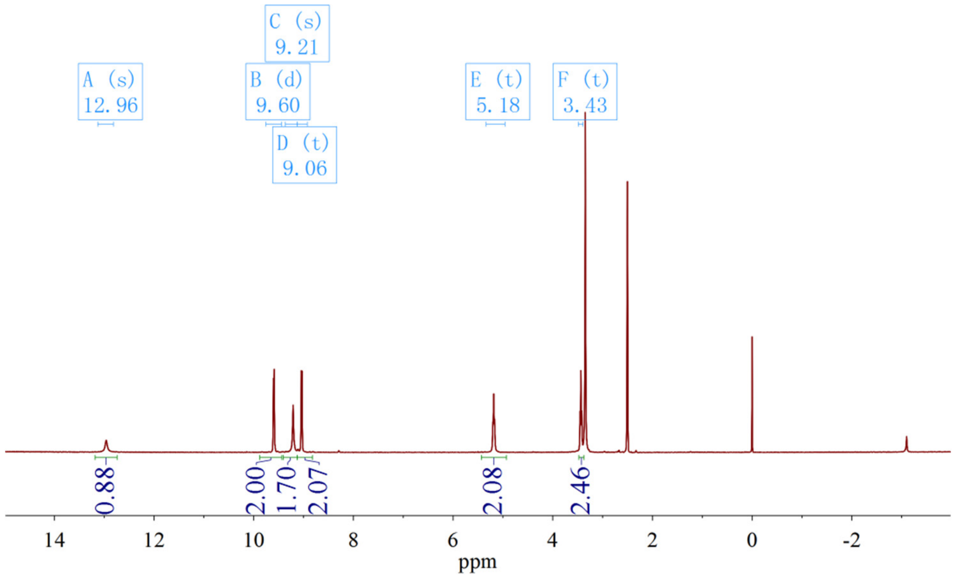

Figure S1. ^{1}H NMR spectra (400 MHz, d6-DMSO) of the TPPOR

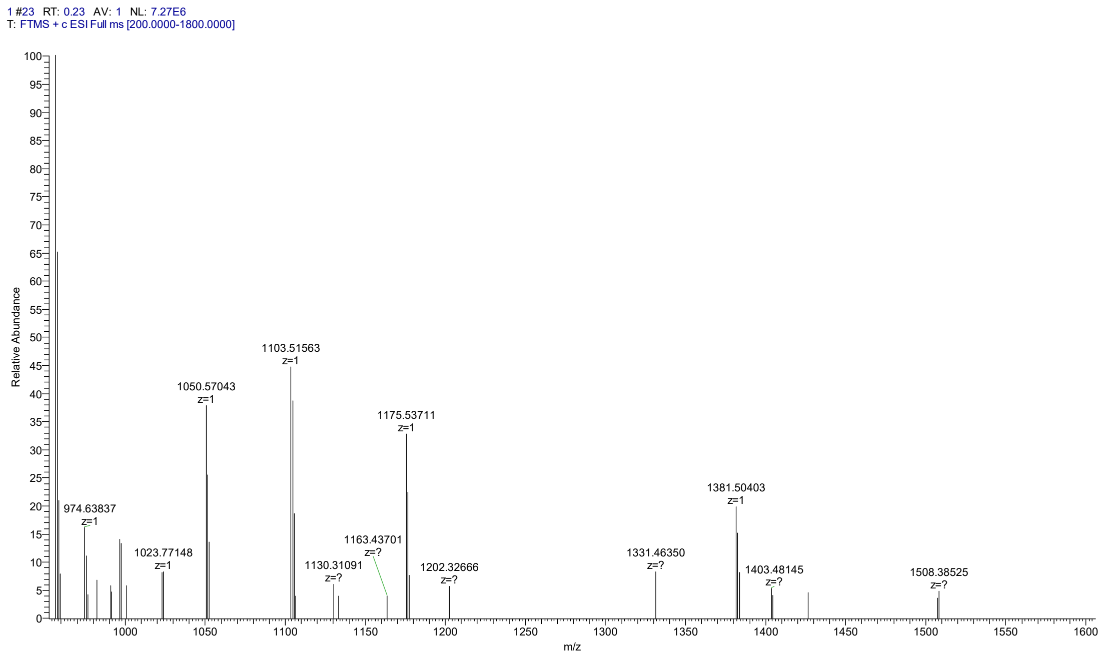

Figure S2. High-resolution mass spectrometry of TPPOR

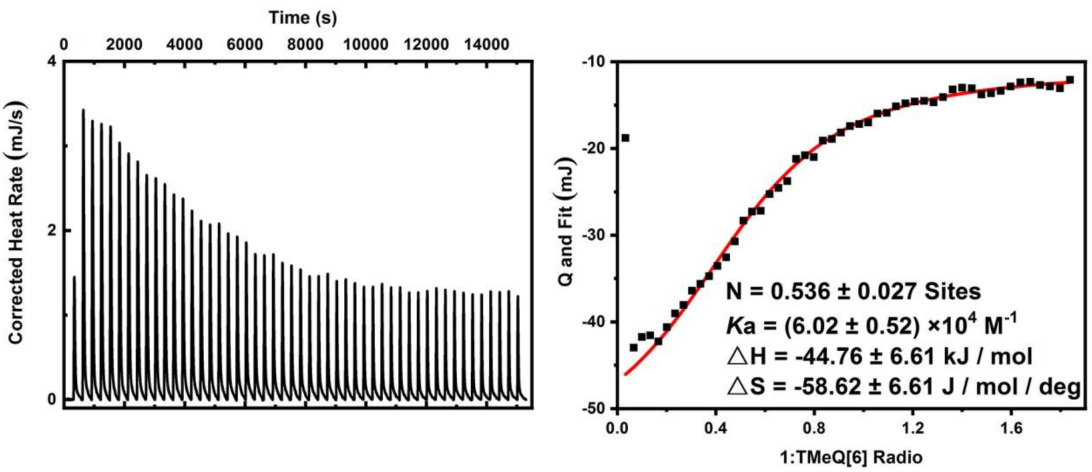

Figure S3. ITC data for the binding interaction between TMeQ[6] and TPPOR at a temperature of 25 °C

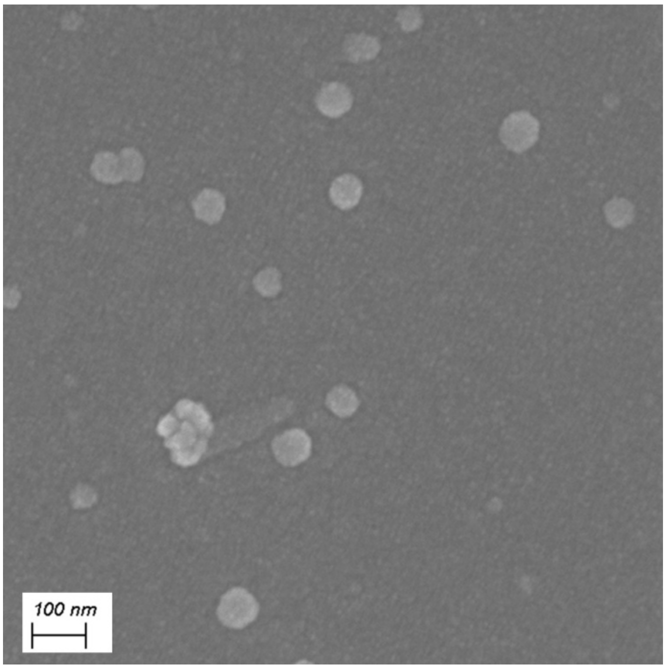

Figure S4. SEM images of TPPOR in aqueous s solution

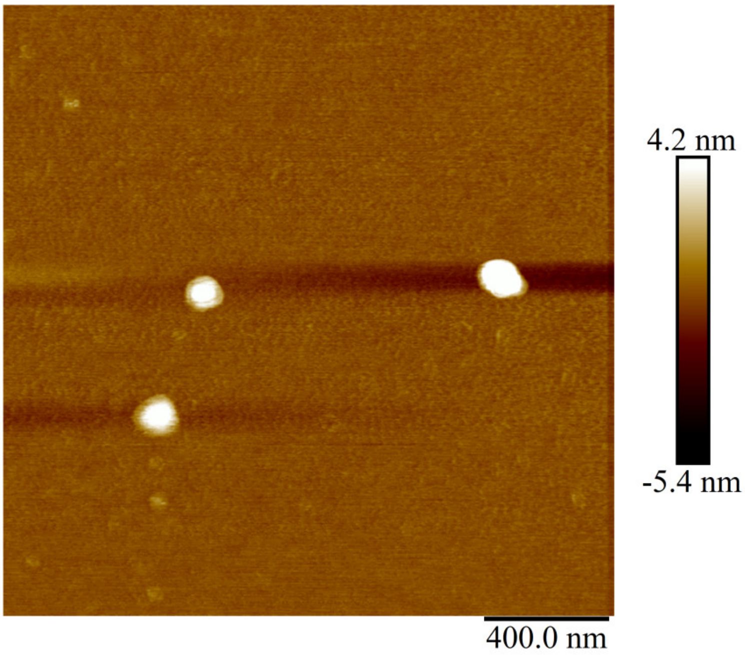

Figure S5. AFM images of TPPOR-2TMeQ[6] in aqueous s solution

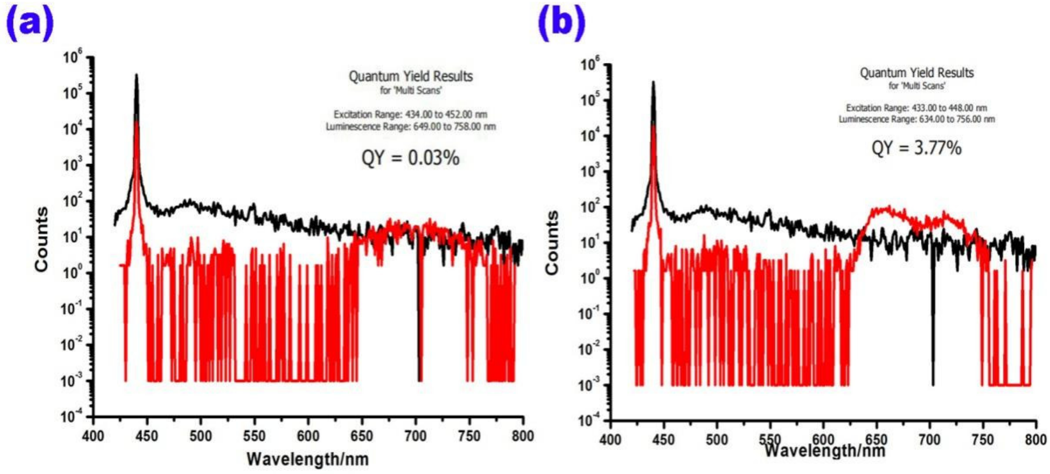

Figure S6. (a) Quantum yield results of TPPOR and (b) TPPOR-2TMeQ[6] (20 µM, pH 7.2). Excitation range: 434 to 452 nm, Luminescence range: 649.00 to 758.00 nm

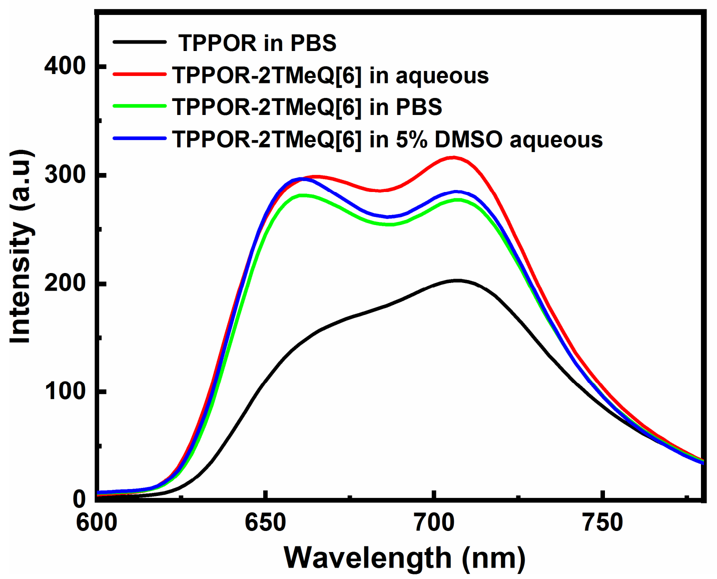

Figure S7. Fluorescence spectra of TPPOR-2TMeQ[6] (20 μM) in various solutions.

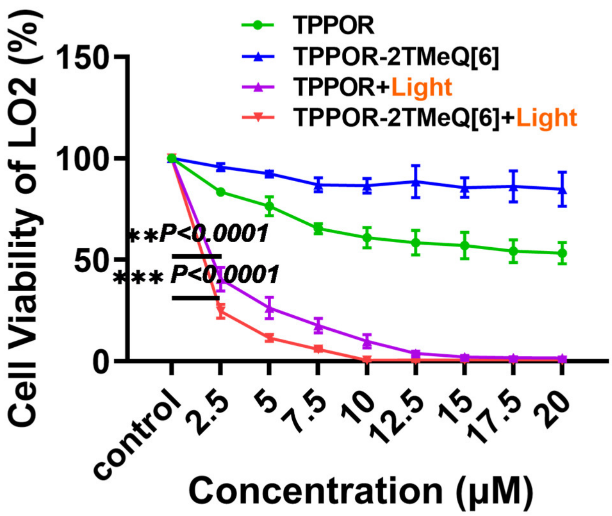

Figure S8. The cell viability of LO2 cells after treatment with different doses of TPPOR and TPPOR-2TMeQ[6] under 520 nm laser irradiation for 3 min (n = 3).

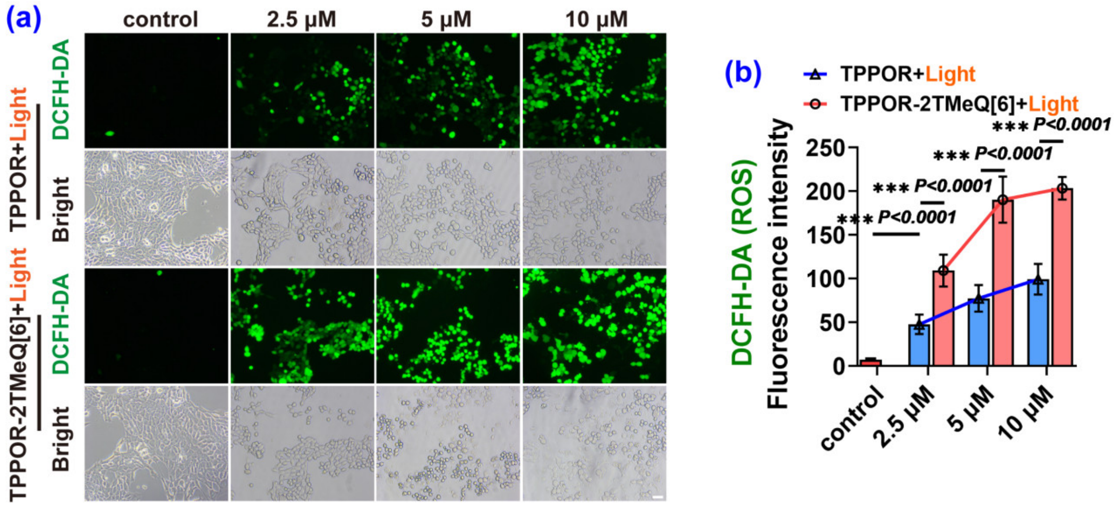

Figure S9. (a) The levels of intracellular ROS in 4T1 cells after treatment with different doses of TPPOR and TPPOR-2TMeQ[6] under 520 nm laser irradiation for 3 min, scale bar = 20 μm, (b) corresponding quantification of ROS (n = 10).
